# Nuclear localization of Newcastle disease virus matrix protein promotes virus replication by affecting viral RNA synthesis and transcription and inhibiting host cell transcription

**DOI:** 10.1186/s13567-019-0640-4

**Published:** 2019-03-20

**Authors:** Zhiqiang Duan, Shanshan Deng, Xinqin Ji, Jiafu Zhao, Chao Yuan, Hongbo Gao

**Affiliations:** 1 Key Laboratory of Animal Genetics, Breeding and Reproduction in The Plateau Mountainous Region, Ministry of Education, Guizhou University, Guiyang, China; 2 College of Animal Science, Guizhou University, Guiyang, China

## Abstract

Nuclear localization of paramyxovirus proteins is crucial for virus life cycle, including the regulation of viral replication and the evasion of host immunity. We previously showed that a recombinant Newcastle disease virus (NDV) with nuclear localization signal mutation in the matrix (M) protein results in a pathotype change and attenuates viral pathogenicity in chickens. However, little is known about the nuclear localization functions of NDV M protein. In this study, the potential functions of the M protein in the nucleus were investigated. We first demonstrate that nuclear localization of the M protein could not only promote the cytopathogenicity of NDV but also increase viral RNA synthesis and transcription efficiency in DF-1 cells. Using microarray analysis, we found that nuclear localization of the M protein might inhibit host cell transcription, represented by numerous up-regulating genes associated with transcriptional repressor activity and down-regulating genes associated with transcriptional activator activity. The role of representative up-regulated gene prospero homeobox 1 (PROX1) and down-regulated gene aryl hydrocarbon receptor (AHR) in the replication of NDV was then evaluated. The results show that siRNA-mediated knockdown of PROX1 or AHR significantly reduced or increased the viral RNA synthesis and viral replication, respectively, demonstrating the important roles of the expression changes of these genes in NDV replication. Together, our findings demonstrate for the first time that nuclear localization of NDV M protein promotes virus replication by affecting viral RNA synthesis and transcription and inhibiting host cell transcription, improving our understanding of the molecular mechanism of NDV replication and pathogenesis.

**Electronic supplementary material:**

The online version of this article (10.1186/s13567-019-0640-4) contains supplementary material, which is available to authorized users.

## Introduction

Paramyxoviruses describe a family of non-segmented negative-sense RNA viruses (NNSV) responsible for significant human and animal diseases, such as measles virus (MeV), mumps virus (MuV), Nipah virus (NiV), Hendra virus (HeV), Sendai virus (SeV), parainfluenza virus types 1–5, and Newcastle disease virus (NDV) [1]. The RNA genomes of paramyxoviruses are 15–19 kb in length and contain six to ten genes that encode six structural viral proteins, including fusion protein (F), attachment protein (HN or H or G), nucleocapsid protein (N or NP), phosphoprotein protein (P), large polymerase protein (L), matrix protein (M) [2, 3]. Of all these proteins, the M protein is the most abundant protein in the virions and forms an outer protein shell around the nucleocapsid, constituting the bridge between the nucleocapsid and viral envelope [4]. Numerous studies have demonstrated that the M protein of most paramyxoviruses is a nucleocytoplasmic shuttling protein [5]. In addition to participating in the assembly and budding of progeny virions at the cell membrane later in infection [6, 7], the M protein is localized in the nucleus early in infection, which may inhibit host cell transcription [5]. Up to now, the detailed functions of M protein in the nucleus has only been clarified in some NNSV such as human respiratory syncytial virus (HRSV) [8], vesicular stomatitis virus (VSV) [9, 10], and MeV [11], but the precise functions of M’s nuclear localization of NDV and other paramyxoviruses remains enigmatic.

Newcastle disease virus, an important member of the paramyxoviruses, is a highly infectious agent of avians that causes substantial economic losses to the poultry industry worldwide [12]. To date, the role of viral F, HN and NP proteins in the replication and pathogenicity of NDV has been extensively studied [13–16], but for the M protein, researchers have always focused on the role of M protein in the formation of NDV virus-like particles [6, 17–19] and the effect of cellular proteins interacting with M on the replication and pathogenicity of NDV [20–24]. However, there is limited information about the nuclear localization functions of NDV M protein. A previous study has shown that NDV M protein enters the nucleus via a bipartite nuclear localization signal (NLS) independently of other viral proteins [25]. In our recent studies, we demonstrated that importin β1 is the nuclear transport receptor of NDV M protein, mediating the nuclear import of M protein by binding its NLS region (^247^KKGKKVIFDKIEEKIRR^263^) through the RanGTP-dependent pathway [22]. Moreover, we also found that a recombinant NDV with NLS mutation (^247^AAGAAVIFDKIEEKIAA^263^) in the M protein (rSS1GFP-M/NLSm) results in a pathotype change of virulent NDV and attenuated viral replication and pathogenicity in SPF chickens [22]. These results clearly indicate that nuclear localization of M protein plays important roles in the replication and pathogenicity of NDV.

In the present study, the parental NDV rSS1GFP and the mutant NDV rSS1GFP-M/NLSm harboring M/NLS mutation were used to investigate the potential functions of M protein in the nucleus. We found that nuclear localization of NDV M protein not only promoted the cytopathogenicity of NDV but also increased viral RNA synthesis and transcription efficiency. Further microarray analysis revealed that nuclear localization of M protein obviously affected cellular binding, catalytic activity, transcription regulator activity, molecular function regulator and transporter activity. Remarkably, nuclear localization of M protein might inhibit host cell transcription, represented by numerous up-regulating genes associated with transcriptional repressor activity and down-regulating genes associated with transcriptional activator activity. In addition, the results of quantitative real time polymerase chain reaction (qRT-PCR) were consistent with those of the microarray results. Moreover, siRNA-mediated knockdown of the selected prospero homeobox 1 (PROX1) (up-regulation gene) or aryl hydrocarbon receptor (AHR) (down-regulation gene) significantly decreased or increased the viral RNA synthesis and viral replication. Overall, our findings revealed that nuclear localization of NDV M protein could promote virus replication by affecting viral RNA synthesis and transcription and inhibiting host cell transcription.

## Materials and methods

### Cells, viruses and antibodies

Chicken embryonic fibroblasts (DF-1) were purchased from the Cell Resource Center of Shanghai Institutes for Biological Sciences of the Chinese Academy of Sciences. BSR-T7/5 cells stably expressing the T7 phage RNA polymerase were a kind gift from Prof. Zhigao Bu (Harbin Veterinary Research Institute, China). DF-1 and were BSR-T7/5 cells were maintained in Dulbecco’s modified Eagle’s medium (DMEM) supplemented with 10% fetal bovine serum (FBS) and antibiotics and were cultured at 37 °C under 5% CO_2_. The parental NDV (rSS1GFP) and the mutant NDV harboring NLS mutation in the M protein (rSS1GFP-M/NLSm) were generated in our previous study [22]. The two viruses were plaque purified three times in DF-1 cells and propagated once in specific-pathogen-free (SPF) embryonated chicken eggs. Primary antibody mouse anti-Tubulin monoclonal antibody (sc-53646), mouse anti-GAPDH monoclonal antibody (sc-66163), mouse anti-Lamin B1 monoclonal antibody (sc-56143), and mouse anti-GFP monoclonal antibody (sc-9996) were purchased from Santa Cruz Biotechnology (USA). Mouse anti-PROX1 monoclonal antibody (ab33219), and rabbit anti-AHR polyclonal antibody (ab84833) were purchased from Abcam (UK).

### Indirect immunofluorescence assay

DF-1 cells grown in 12-well plates were infected with NDV strain rSS1GFP or rSS1GFP-M/NLSm at a multiplicity of infection (MOI) of 5 and then prepared for immunofluorescence analysis at 6, 12 and 18 hours post-infection (hpi). At the indicated time, cells were rinsed with phosphate-buffered saline (PBS), fixed with 4% paraformaldehyde for 15 min, and then permeabilized with 0.25% Triton X-100 for 5 min. Cells were rinsed with PBS and blocked with 10% FBS in PBS for 30 min, and then incubated with mouse anti-M polyclonal antibody [21] diluted in PBS containing 10% FBS for 1 h. After three washes with PBS, the cells were incubated with Alexa Fluor 488 goat anti-mouse immunoglobulin G antibody (Invitrogen) for 1 h. Cells were counterstained with DAPI (Sigma) to detect the nuclei. Images were captured with a fluorescence microscope and processed with Adobe Photoshop 7.0 software.

### Syncytia assays

DF-1 cells were grown to 80% confluence in 6-well plates and then co-transfected with plasmids pCI-F (1 μg), pCI-HN (1 μg), and pCI-M (1 μg) or pCI-M/NLSm (1 μg) using FuGENE HD Transfection Reagent (Promega) according to the manufacturer’s instructions. Cells co-transfected with pCI-F and pCI-HN were used as controls. After 3 days post-transfection, cells were rinsed with PBS, fixed with cold methanol and then stained with Giemsa. The number of syncytia (cells containing more than three nuclei) was counted in ten random areas of the well.

### Minigenome assays

To evaluate the effect of M’s nuclear localization on viral RNA synthesis, the minigenome assays were performed as described previously [26]. Briefly, BSR-T7/5 cells in six-well plates were transfected with pTVT-TGL (1 μg), pCI-NP (1 μg), pCI-P (0.5 μg), pCI-L (0.5 μg), pCI-M (1 μg) or pCI-M/NLSm (1 μg) using FuGENE HD Transfection Reagent. For negative controls, pCI-M or pCI-M/NLSm was replaced by empty vector pCI-neo in the minigenome system to normalize the total amount of transfected DNA. To detect minigenome-specific RNA and protein levels, the cells were harvested at 12, 24, 36 and 48 hours post-transfection (hpt) for real-time PCR or Western blotting analysis. All assays were repeated at least three times.

### Quantification of minigenomic or viral RNA synthesis by qRT-PCR

Plasmids-transfected BSR-T7/5 cells or viruses-infected DF-1 cells were collected and then treated with TRIzol reagent (Invitrogen) according to the manufacturer’s instructions. Total RNA was extracted and reverse-transcribed (2 μg per sample) as described previously [27]. Quantification of minigenomic RNA synthesis [27] and viral RNA synthesis [16] by qRT-PCR was performed as previously described, respectively. In addition, primers qGFP-F (5′-CGACAAGCAGAAGAACGGCATCA-3′) and qGFP-R (5′-GGACTGGGTGCTCAGGTAGTGGTT -3′) were used to quantify the GFP gene. qRT-PCR experiments were performed using SYBR Premix Ex Taq (TaKaRa, Japan) according to the manufacturer’s protocol. All the reactions were performed in a 10 μL volume containing 5 μL of 2× SYBR Premix Ex Taq, 200 nM of each primer, and 0.2 μL ROX reference DyeII. The cycling parameters were 1 cycle at 94 °C for 30 s, followed by 40 cycles at 94 °C for 5 s, 60 °C for 10 s, and 60 °C for 15 s. The threshold cycle 2^−∆∆CT^ method was used to determine the fold change of gene expression levels.

### Cell fractionation and Western blotting

DF-1 cells infected with rSS1GFP and rSS1GFP-M/NLSm were washed twice with PBS and fractionated for extraction of the nuclear and cytoplasmic proteins following the manufacturer’s instructions (Beyotime Biotechnology, China). The nuclear or cytoplasmic proteins were resolved by sodium dodecyl sulfate polyacrylamide gel electrophoresis (SDS-PAGE), and then transferred onto a polyvinylidene difluoride (PVDF) membrane. The membranes were blocked for 1 h at room temperature with 5% skim milk in Tris-buffered saline-Tween (TBST) (20 mM Tris–HCl [pH 7.4], 137 mM NaCl, and 0.1% Tween-20) and then incubated overnight at 4 °C with the primary polyclonal antibody against M. The blots were washed three times in TBST buffer and incubated for 1 h at room temperature with horseradish peroxidase-conjugated anti-mouse IgG. Mouse anti-Lamin B1 or anti-Tubulin monoclonal antibody was used as an internal standard. The relative levels of the M protein to control Lamin B1 or Tubulin expression were determined by densitometry using Bandscan 5.0 software.

### Microarray analysis

Transcriptional profiles were determined by microarray analysis of nuclear RNA isolated from the nucleus of rSS1GFP- or rSS1GFP-M/NLSm- or mock-infected cells at 6, 12 and 18 hpi. Total RNA were extracted from both infected and non-infected DF-1 cells using TRIzol reagent (Invitrogen), and nuclear RNA was further purified by Cytoplasmic and Nuclear RNA Purification Kit (Norgen, Canada) following the manufacturer’s protocol. The nuclear RNA was then amplified, labeled and purified using a GeneChip 3′ IVT Express Kit (Affymetrix) to obtain biotin-labeled cRNA. Array hybridization and washing were performed using GeneChip^®^ Hybridization and the Wash and Stain Kit (Affymetrix) in the Hybridization Oven 645 (Affymetrix) and Fluidics Station 450 (Affymetrix). Slides were scanned using a GeneChip Scanner 3000 and raw data were normalized using the MAS 5.0 algorithm, Gene Spring Software 11.0 (Annoroad Gene Technology).

*T*-test and significant analysis of microarray were performed to identify the genes that had significantly different expression levels (*P* < 0.05 and > 2-fold change) with infection compared to levels in mock infections. For bio-function and pathway analysis, files containing significantly differentially expressed (SDE) genes were uploaded into IPA platform (Ingenuity Systems, CA, USA). Fisher’s exact test was used to determine the probability that each biological function assigned to the genes within each data set was due to chance alone.

### Analysis of gene expression by qRT-PCR

Based on the microarray results, 12 genes were selected for the qRT-PCR analysis using SYBR Premix Ex Taq Kit (TaKaRa, Japan) according to the manufacturer’s instructions. The sequences of the selected SDE genes were searched in GenBank, and primers (Table 1) for qRT-PCR were designed based on the target sequences using Primer Premier 5.0 Software. All the reactions were performed in a 10 μL volume containing 5 μL of 2× SYBR Premix Ex Taq, 200 nM of each primer, and 0.2 μL ROX reference DyeII. The cycling parameters were 1 cycle at 95 °C for 5 s followed by 40 cycles at 95 °C for 5 s and 60 °C for 31 s. One cycle of melting curve analysis was added for all reactions to verify product specificity. The relative levels of gene expression were normalized to that of the GAPDH gene. The threshold cycle 2^−△△CT^ method was used to determine the fold change of gene expression levels.

**Table 1:** **Quantitative real-time PCR primers used in this study**

Gene name	Forward primer (5′ → 3′)	Reverse primer (5′ → 3′)	GenBank no.
NDV M	CTGTGCTTGTGAAGGCGAGAGGT	TGGGGAGAGGCATTTGCTATAGGAT	KP742770
PROX1	GCAGGTCAGACAACGAGATGTGC	AGGAATTTGGCCCTTGGTCCTTC	NM_001005616
AHRR	TCATCACGAAGGTGCAGCTTTCG	TGCTGTTGGGCAACAACATATTCTG	HQ340610
RARA	AGAGCACCAGCTCAGAGGAGATCGT	ACCCCATAGTGGTACCCGGAGGAT	NM_204536
ZEB2	CAGCGACACAGCCATTATTTACCCC	AGTTCCAGGTGGCAGGTCGTTTTC	NM_001318466
PAIP2	AGCCCAAGCATCATCAGTGAAGATG	CCACAACTCCTCTTCAATCTGCCTG	XM_025154840
RUNX2	ACCACAGAGCCATAAAGGTGACG	GGGACCCCTACTCTCATACTGGGA	NM_204128
AHR	GCGTAACATGAAGTTGCCCTTCATG	GTTTTGCCTCCTTTTCCTGTGGTG	NM_204118
HOXB4	GTTCCCACCCTGTGAAGAGTATTCC	TCATGTTGGAAAGTGCTCTCTCGC	NM_205293
NF1A	AACCCGAGGTAAAGCAGAAATGG	GAGGCTTTTTTCCTGTGACTGTGAG	NM_205273
IRF2	GAGCCAGTTGAATCATCTTTTGGGA	CAAGGTGCGGCTGTCCTACAACTA	NM_205196
TAF9	TGATGGCGCAGATCCTGAAGGATA	GTAAATCTTCGCGTCCTCCAGGATG	NM_001277796
LHX2	GGAGATTTTCGGTGCAGAGATGTGC	GTGGTCAGCATCTTGTTGCAAGTGG	NM_204889
GAPDH	TCAAGGCTGAGAACGGGAAACTTG	TGGACTCCACAACATACTCAGCACC	NM_204305

### Small interfering RNA (siRNA) assays

Small interfering RNA-mediated RNA interference was used to knock down ATF6 and AHR in BSR-T7/5 cells. Mouse PROX1 siRNA (sc-152489), AHR siRNA (sc-29655) and Control siRNA (sc-37007) were purchased from Santa Cruz Biotechnology Company. For transfection with the siRNA against PROX1 or AHR, low-passage BSR-T7/5 cells grown in 6-well plates were transfected with 30 pmol siRNA using 9 μL Lipofectamine RNAiMAX (Thermo Fisher Scientific, USA) in Opti-MEM medium (Thermo Fisher Scientific). The knockdown efficiency was measured by examining endogenous protein expression by Western blotting analysis after 48 h transfection. To study the effect of PROX1 or AHR knockdown on the replication of NDV, the viruses were used to infect PROX1 or AHR siRNA-treated BSR-T7/5 cells at an MOI of 5. The detection of viral RNA synthesis and viral replication in BSR-T7/5 cells were examined as described above.

### Statistical analysis

Differences in the expression level of genes and virus titers between cells infected with rSS1GFP and rSS1GFP-M/NLSm were analyzed using SPSS Statistics software. The independent-samples *t* test was used for data analysis. A *P*-value of < 0.05 was considered significant. *P*-values are indicated by asterisks (*P < 0.05, **P < 0.01, ***P < 0.001).

## Results

### Nuclear localization of M protein promotes the cytopathogenicity of NDV

**Figure 1:**
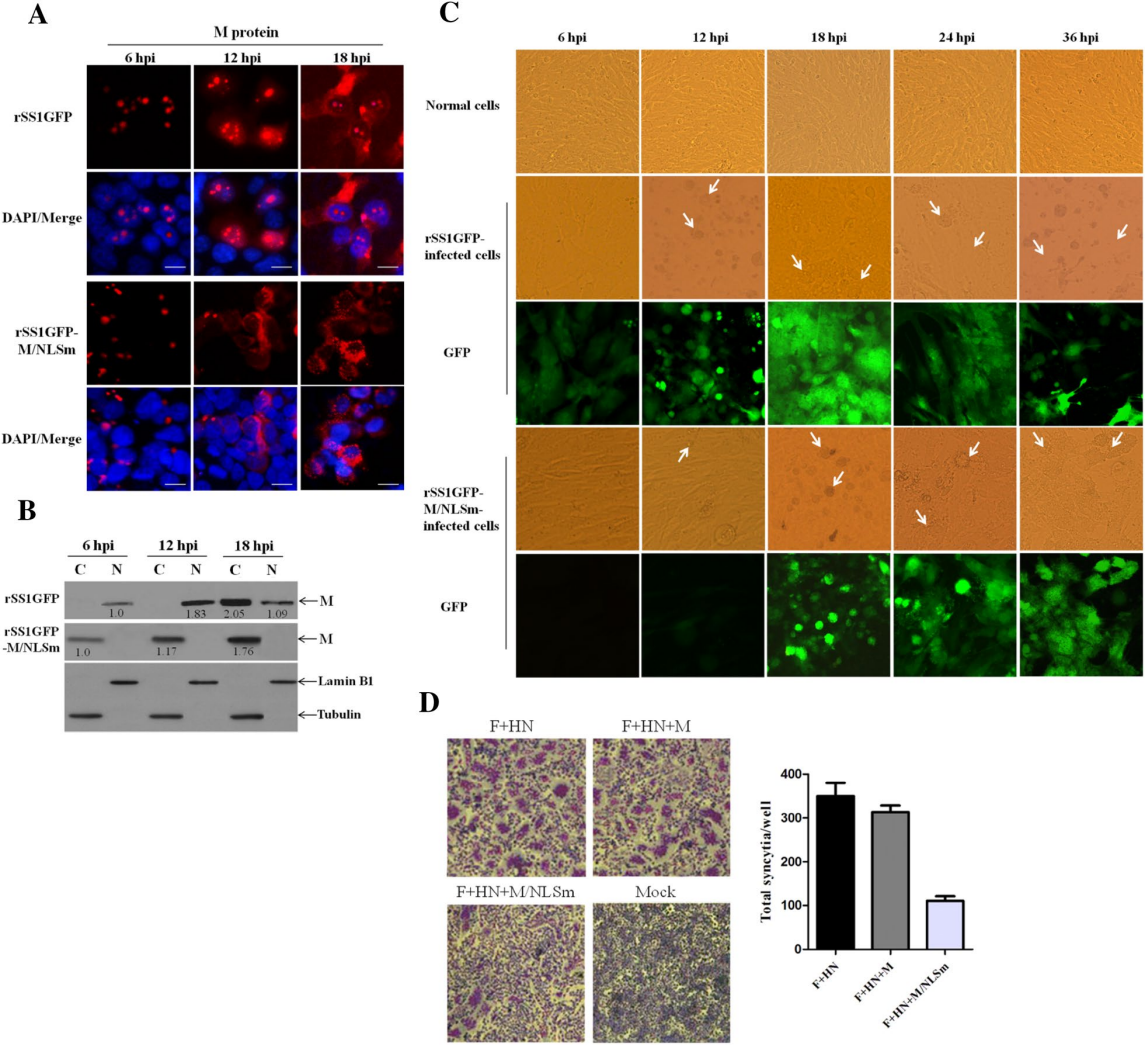
**Nuclear localization of M protein promotes the cytopathogenicity of NDV.** The subcellular localizations of M protein in DF-1 cells infected with rSS1GFP or rSS1GFP-M/NLSm were examined by immunofluorescence staining (**A**) and Western blotting (**B**). The relative levels of the M protein were compared with the control Lamin B1 or Tubulin expression. N represents the nucleus and C represents the cytoplasm. **C** The CPE and GFP expression were observed in rSS1GFP- or rSS1GFP-M/NLSm-infected DF-1 cells at the indicated time points. Original magnification was 1 × 200. **D** The syncytium formation in plasmids-transfected DF-1 cells. The number of syncytia (cells containing more than three nuclei) was counted in 10 random areas of the well (mean ± SD).

We previously reported that NLS mutation in NDV M protein not only disrupted the nuclear localization of M protein, but also impaired the replication efficiency and plaque formation ability of NDV [22]. To learn more about the effect of NLS mutation on the dynamic changes of the subcellular localization of M, we compared the localization of the M protein in rSS1GFP- and rSS1GFP-M/NLSm-infected cells at different time points. The results show that the M protein of rSS1GFP was primarily localized in the nucleolus followed by a discrete punctuate staining pattern at 6 hpi, and then was found in the largest concentration in the nucleus and nucleolus at 12 hpi; whereas the M protein was distributed diffusely in the cytoplasm, with some still localized in the nucleolus at 18 hpi (Figure 1A). By contrast, the M protein of rSS1GFP-M/NLSm accumulated around the nucleus at 6 hpi, and then distributed exclusively in the cytoplasm at 12 and 18 hpi (Figure 1A). In addition, the intracellular localization of rSS1GFP and rSS1GFP-M/NLSm M protein detected by Western blotting was consistent with the immunofluorescence analysis (Figure 1B). Next, the cytopathic effect (CPE) and the expression of green fluorescent protein (GFP) in virus-infected cells were evaluated. We found that the CPE and GFP expression in rSS1GFP-infected cells started early at 6 hpi and the extensive CPE and GFP expression appeared at 18 hpi, and then the cell monolayer was absolutely destroyed at 36 hpi (Figure 1C). However, the slight CPE and GFP expression in rSS1GFP-M/NLSm infected cells started at 12 hpi and the cell monolayer was still existent at 36 hpi (Figure 1C), demonstrating that rSS1GFP-M/NLSm induced much slighter CPE and GFP expression than that of rSS1GFP. Meanwhile, the syncytia assays show that co-expression of F, HN and M/NLSm caused a marked reduction in cell–cell fusion when compared to co-expression of F and HN or F, HN and M (Figure 1D), indicating that M’s cytoplasmic localization affected the amount of cell–cell fusion. Therefore, these results confirm that nuclear localization of M protein could promote the cytopathogenicity of NDV.

### Nuclear localization of M protein affects viral RNA synthesis and transcription

**Figure 2:**
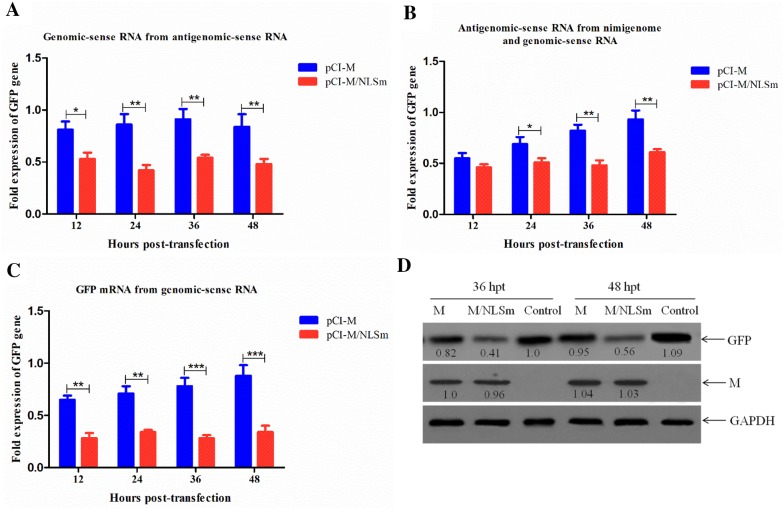
**Quantitative analysis of genomic RNA, antigenomic RNA, mRNA and protein in a minigenomic assay.** Relative fold expression of genomic RNA (**A**), antigenomic RNA (**B**), and mRNA (**C**) in the minigenome system caused by M or M/NLSm were measured by qRT-PCR. Error bars represent standard deviations (mean ± SD) (*P < 0.05; **P < 0.01; ***P < 0.001 compared to the value of negative control). **D** Expression of GFP in the minigenome system caused by M or M/NLSm was detected by Western blotting at 36 and 48 hpt. The relative levels of the M and GFP proteins were compared with the control GAPDH expression.

To investigate the role of M’s nuclear localization in viral RNA synthesis and transcription, an NDV minigenome assay was first performed using GFP as a reporter gene (Additional file 1). The expression of GFP genomic RNA, antigenomic RNA and mRNA was quantified by qRT-PCR to represent the viral RNA synthesis and transcription. The results show that the relative expression level of GFP genomic RNA was decreased in the presence of M/NLSm at different time points in comparison to the presence of M (Figure 2A). Although the relative GFP antigenomic RNA had no significant change at 12 hpt, it shows obviously reduced expression levels in the subsequent time points (Figure 2B). Consistent with the relative expression levels of GFP genomic RNA, the relative GFP mRNA levels significantly declined in the presence of M/NLSm, especially at 36 hpt and 48 hpt (*P* < 0.001) (Figure 2C). Moreover, Western blotting analysis also shows that the GFP expression levels in M/NLSm-cell lysates were much lower than that in M-cell lysates at 36 hpt and 48 hpt (Figure 2D).

**Figure 3:**
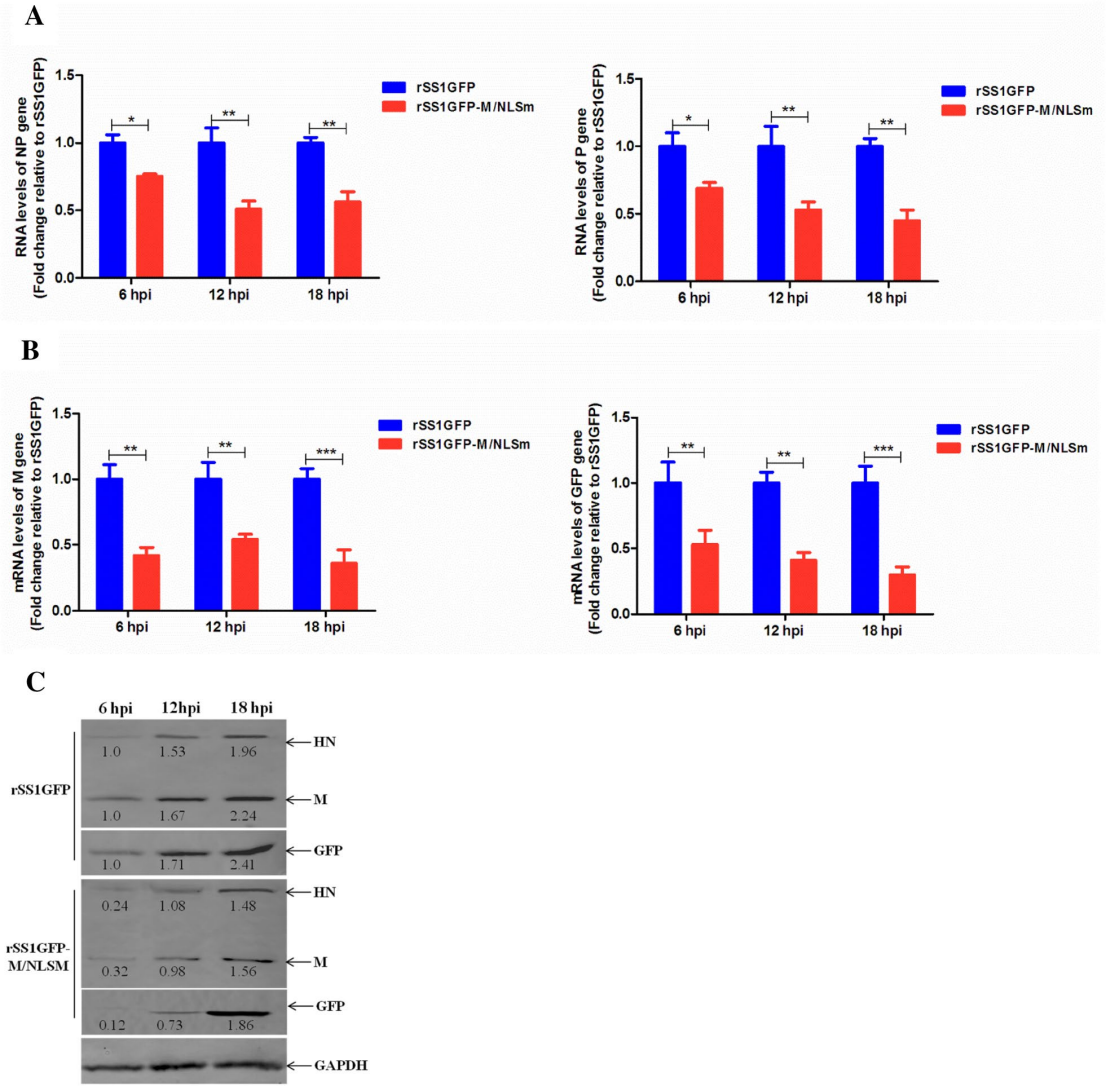
**Comparison of the viral RNA synthesis and transcription in DF-1 cells. A** The viral RNA synthesis corresponding to the NP and P genes and **B** viral transcription corresponding to the M and GFP genes in rSS1GFP- and rSS1GFP-M/NLSm-infected cells were detected by qRT-PCR. Error bars represent standard deviations (mean ± SD) (*P < 0.05; **P < 0.01; ***P < 0.001 compared to the value of rSS1GFP-M/NLSm). **C** The expression levels of M, HN and GFP proteins in rSS1GFP- and rSS1GFP-M/NLSm-infected cells were examined by Western blotting. The relative levels of the M, HN and GFP proteins were compared with the control GAPDH expression.

To further verify whether the disruption of M’s nuclear localization will affect viral RNA synthesis and transcription, we analyzed the RNA levels of NP and P genes and the mRNA levels of M and GFP genes in rSS1GFP- and rSS1GFP-M/NLSm-infected cells. As shown in Figure 3A, there were statistically significant differences in the relative RNA levels (corresponding to the NP and P genes) between cells infected with rSS1GFP and rSS1GFP-M/NLSm at 6 hpi (*P* < 0.05). Moreover, the relative levels of viral RNA in rSS1GFP-M/NLSm-infected cells were more decreased than that in rSS1GFP-infected cells at 12 and 18 hpi (*P* < 0.01) (Figure 3A). On the contrary, we found that the relative mRNA levels of M and GFP genes in rSS1GFP-M/NLSm-infected cells were also much lower than that in rSS1GFP-infected cells at 6 and 12 hpi (*P* < 0.01), and significantly lower at 18 hpi (*P* < 0.001) (Figure 3B). Meanwhile, the expression levels of HN, M and GFP proteins were relatively decreased during the course of rSS1GFP-M/NLSm infection (Figure 3C), suggesting that cytoplasmic M protein could result in the reduced viral transcription. Collectively, these results indicate that nuclear localization of M protein affected the viral RNA synthesis and transcription, which would benefit NDV replication.

### Nuclear localization of M protein induces robust host response by microarray analysis

**Figure 4:**
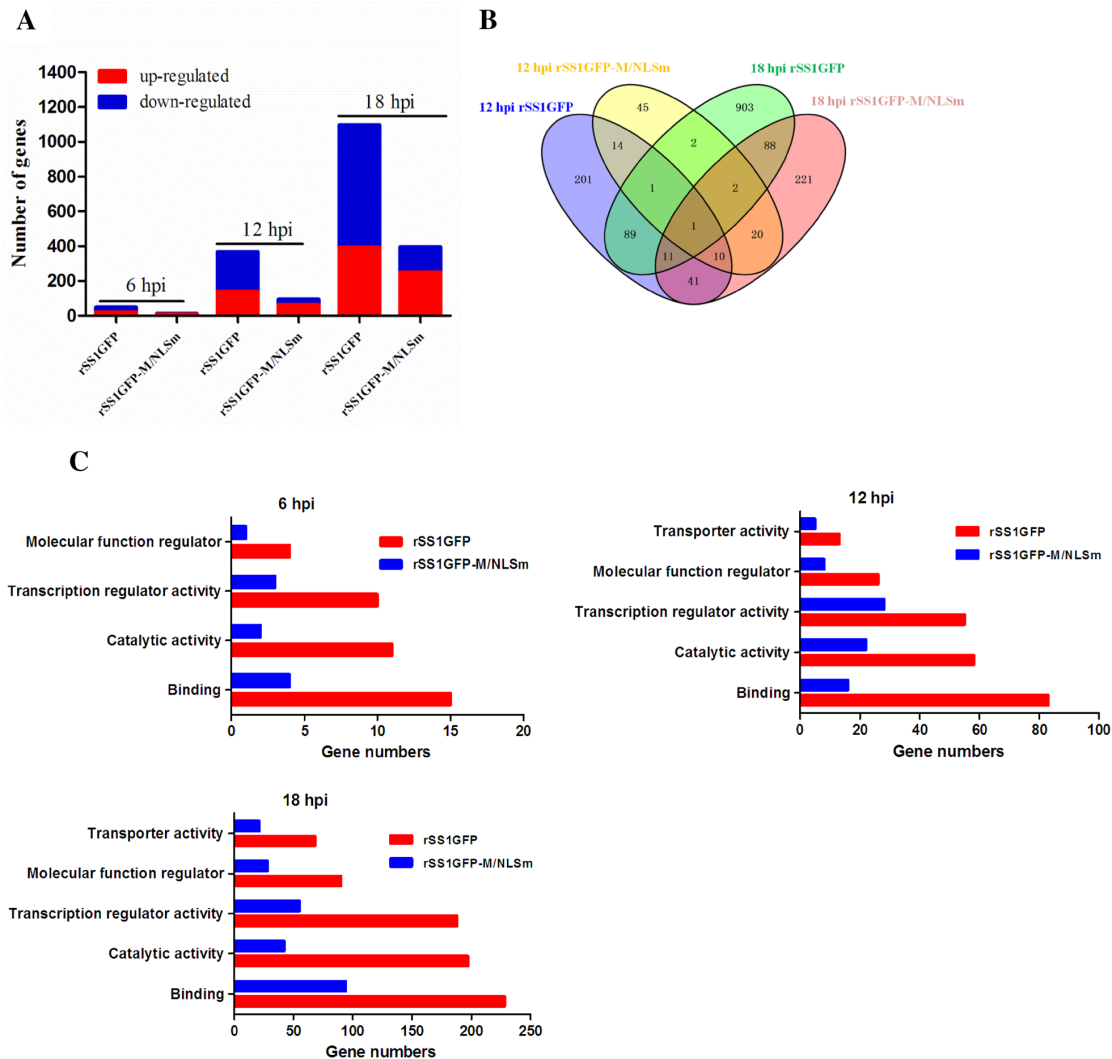
**Microarray analysis of gene expression in DF-1 cells infected with rSS1GFP and rSS1GFP-M/NLSm. A** Numbers of different expressed genes during infection with rSS1GFP or rSS1GFP-M/NLSm virus relative to mock infection at 6, 12 and 18 hpi (*P* < 0.05, fold change > 2). **B** Venn diagram showing the distribution of different expressed genes during infection with rSS1GFP or rSS1GFP-M/NLSm virus at 12 and 18 hpi. **C** Functional categories of different expressed genes in cells infected with rSS1GFP or rSS1GFP-M/NLSm virus at 6, 12 and 18 hpi.

To understand the contribution of M’s nuclear localization to NDV replication, we compared transcriptional profiles of host genes in DF-1 cells infected with rSS1GFP or rSS1GFP-M/NLSm by microarray analysis. A total of 50, 368 and 1097 genes were obtained with significantly differential expression (SDE) levels (*P* < 0.05 and > 2-fold change) during rSS1GFP infection at 6, 12 and 18 hpi, respectively, whereas the corresponding gene numbers were 12, 95 and 395 for rSS1GFP-M/NLSm at the indicated time points (Figure 4A). In addition, a Venn diagram summarizing the distribution of SDE genes revealed that only 26 and 102 genes were shared by rSS1GFP and rSS1GFP-M/NLSm at 12 hpi and 18 hpi, respectively (Figure 4B). Biological function analysis using Ingenuity Pathway Analysis (IPA) platform demonstrates that the remarkable differences in gene expression between rSS1GFP and rSS1GFP-M/NLSm were mainly associated with binding, catalytic activity, transcription regulator activity, molecular function regulator and transporter activity (Figure 4C). Canonical pathway analysis shows that rSS1GFP preferred to modulate the signaling pathways associated with virus infection, while rSS1GFP-M/NLSm tended to regulate metabolism pathways related to cellular function and maintenance (Table 2). Together, these results indicate that rSS1GFP elicited a more potent host response than rSS1GFP-M/NLSm.

**Table 2:** **Top five IPA canonical pathways modulated by rSS1GFP and rSS1GFP-M/NLSm**

Time points (hpi)	Virus	IPA canonical pathway^a^	*P* value	Gene count^b^
6	rSS1GFP	Toll-like receptor signaling pathway	3.62E−04	3/10
		RIG-I-like receptor signaling pathway	4.15E−04	1/8
		NF-kappa B signaling pathway	2.18E−03	2/8
		Hepatitis C	3.70E−03	2/12
		TGF-β signaling pathway	4.53E−03	1/6
	rSS1GFP-M/NLSm	Apelin signaling pathway	2.15E−03	0/2
		Hippo signaling pathway	3.44E−03	1/5
		Pyrimidine metabolism	1.35E−02	0/3
12	rSS1GFP	Cytokine–cytokine receptor interaction	8.93E−10	8/12
		HTLV-1 infection	2.06E−9	9/21
		Kaposi sarcoma-associated herpesvirus infection	6.38E−9	10/32
		Transcriptional misregulation in cancer	2.28E−8	4/7
		TNF signaling pathway	3.80E−7	6/16
	rSS1GFP-M/NLSm	Thyroid hormone signaling pathway	3.63E−5	3/13
		Biosynthesis of amino acids	4.96E−5	1/8
		Rap1 signaling pathway	2.11E−4	3/8
		Glycine, serine and threonine metabolism	2.25E−3	2/10
		Non-small cell lung cancer	2.63E−3	1/9
18	rSS1GFP	MAPK signaling pathway	1.01E−11	21/42
		TNF signaling pathway	2.70E−10	12/36
		MAPK signaling pathway	6.99E−9	9/23
		Human papillomavirus infection	1.38E−8	10/21
		Transcriptional misregulation in cancer	3.31E−8	15/38
	rSS1GFP-M/NLSm	DNA replication	1.09E−4	6/44
		Neurotrophin signaling pathway	1.51E−3	4/23
		Cellular senescence	2.36E−3	3/26
		Insulin resistance	4.15E−2	2/15
		Caffeine metabolism	7.38E−2	2/12

^a^Top 5 IPA significant canonical pathways are represented (cutoff for significance, *P* < 0.05). Only three pathways were detected in rSS1GFP-M/NLSm-infected cells at 6 hpi.

^b^Numbers of genes are provided as X/Y, where Y is the total number of genes in the pathway and X is the amount of differentially expressed genes in response to viral infection.

### Nuclear localization of M protein inhibits host cell transcription

**Figure 5:**
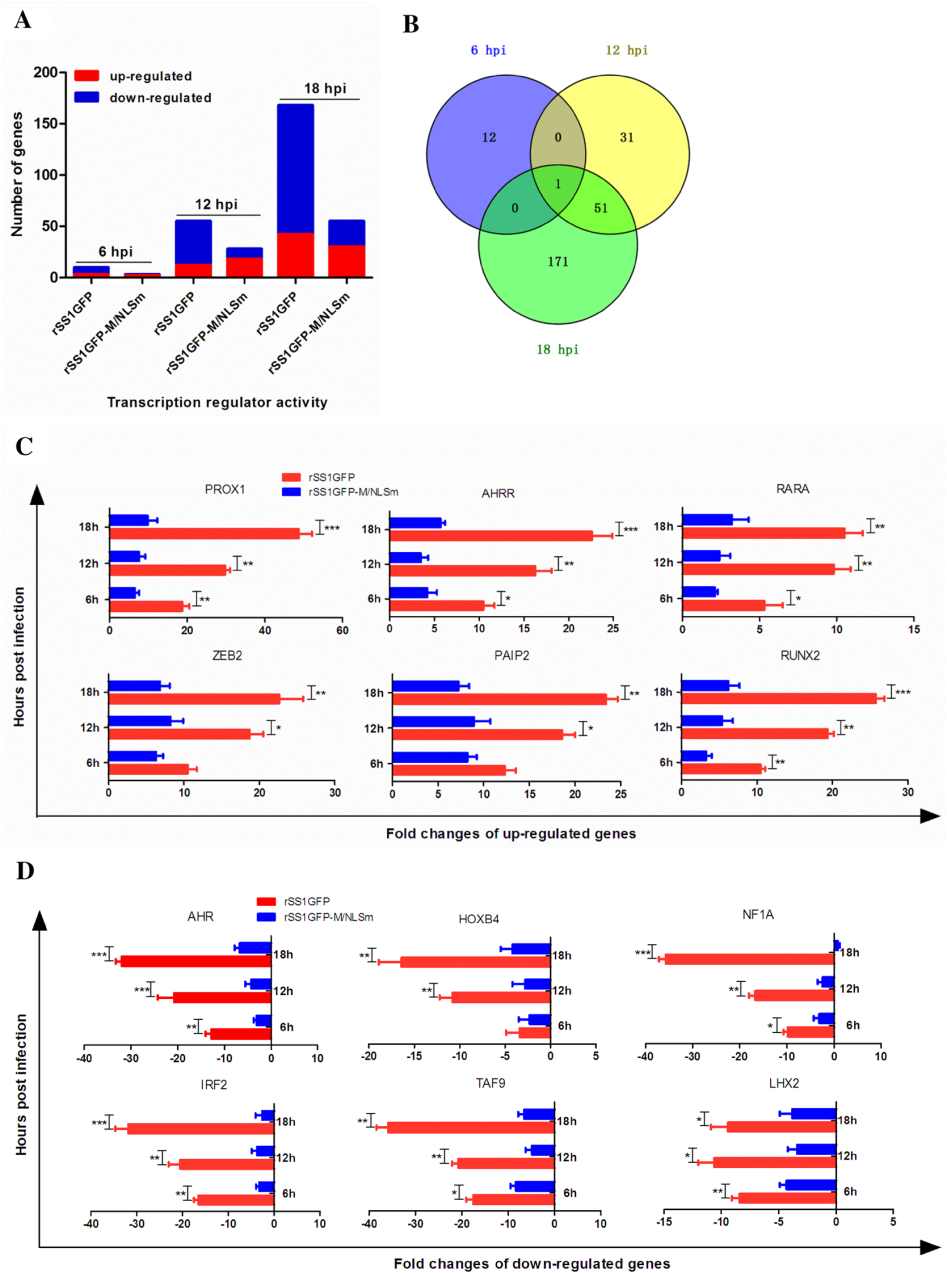
**Analysis of the expression levels of transcription regulator activity related genes in DF-1 cells. A** Numbers of different expressed genes associated with transcription regulator activity during infection with rSS1GFP or rSS1GFP-M/NLSm virus relative to mock infection at 6, 12 and 18 hpi (*P* < 0.05, fold change > 2). **B** Venn diagram showing the distribution of different expressed genes associated with transcription regulator activity during infection with rSS1GFP or rSS1GFP-M/NLSm virus at 6, 12 and 18 hpi. The expression levels of six selected up-regulated genes (**C**) and six down-regulated genes (**D**) associated with transcription regulator activity were verified by qRT-PCR. Error bars represent standard deviations (mean ± SD) (*P < 0.05; **P < 0.01; ***P < 0.001 compared to the value of rSS1GFP-M/NLSm).

Several studies have demonstrated that the M protein of NNSV including HRSV, VSV and MeV can inhibit host cell transcription in various ways [8–11, 28–30]. To determine whether the NDV M protein has the ability to inhibit host cell transcription, we mainly focused on the expression profiles of transcription regulator activity-related genes in the results of microarray analysis. A total of 233 and 86 SDE genes associated with transcription regulator activity were identified in DF-1 cells infected with rSS1GFP and rSS1GFP-M/NLSm (Figure 5A), respectively. Meanwhile, the number of SDE genes in rSS1GFP-infected cells mainly showed a tendency of down-regulation (Figure 5A). However, only one common gene was found at 6, 12 and 18 hpi in the virus-infected cells using a Venn diagram (Figure 5B). A further analysis of the top 5 up-regulated and down-regulated SDE genes in rSS1GFP-infected cells shows that the up-regulated SDE genes were mostly associated with the function of transcription repressor activity and negative regulation of transcription (Table 3), while the down-regulated SDE genes were mainly involved in DNA binding or RNA polymerase II transcription factor activity and transcriptional activator activity (Table 4).

**Table 3:** **Top 5 up-regulated SDE genes associated with the transcription regulator activity in virus-infected cells**

Time (hpi)	Gene symbol	Gene description	rSS1GFP		rSS1GFP-M/NLSm		Molecular function
			FC^a^	*P* value	FC	*P* value	
6	ZEB2	Zinc finger E-box binding homeobox 2	6.5	1.21E−05	1.2	2.30E−02	Transcriptional repressor activity
	RARA	Retinoic acid receptor alpha	5.3	6.40E−06	0.8	1.12E−03	Transcription corepressor activity
	PROX1	Prospero homeobox 1	3.2	4.00E−04	0.4	1.51E−02	Negative regulation of transcription
12	AHRR	Aryl hydrocarbon receptor repressor	10.2	4.21E−08	2.3	3.32E−03	Protein heterodimerization activity
	ETS2	ETS proto-oncogene 2	8.4	3.82E−07	1.4	2.12E−02	Transcription repressor activity
	KLF11	Kruppel like factor 11	7.6	1.09E−07	3.6	5.16E−04	Negative regulation of transcription
	HDAC9	Histone deacetylase 9	7.1	2.67E−06	2.7	4.11E−02	Transcription corepressor activity
	SAMD4A	Sterile alpha motif domain containing 4A	5.8	3.23E−06	0.6	1.15E−02	Transcription repressor activity
18	PROX1	Prospero homeobox 1	9.6	4.12E−07	2.2	5.43E−03	Negative regulation of transcription
	PAIP2	Poly(A) binding protein interacting protein 2	8.3	2.54E−05	1.8	2.80E−02	Transcription repressor activity
	ETV3	ETS variant 3	6.4	3.81E−06	3.1	4.78E−04	Repressing transcription factor binding
	RUNX2	Runt related transcription factor 2	6.2	2.41E−03	1.4	3.05E−03	Transcription corepressor activity
	CCAR1	Cell division cycle and apoptosis regulator 1	5.8	1.22E−04	2.0	1.63E−02	Transcription corepressor activity

^a^FC: fold change in expression levels relative to those of uninfected cells. Only three SDE genes were found in rSS1GFP -infected cells at 6 hpi.

**Table 4:** **Top 5 down-regulated SDE genes associated with the transcription regulator activity in virus-infected cells**

Time (hpi)	Gene symbol	Gene description	rSS1GFP		rSS1GFP-M/NLSm		Molecular function
			FC^a^	*P* value	FC	*P* value	
6	AHR	aryl hydrocarbon receptor	−4.1	2.29E−05	−1.1	8.39E−04	DNA binding transcription factor activity
	NF1A	Nuclear factor I A	−3.5	2.04E−04	−0.8	2.43E−03	RNA polymerase II transcription factor activity
	TFCP2L1	Transcription factor CP2 like 1	−3.0	3.27E−06	−0.4	1.66E−02	RNA polymerase II regulatory region sequence-specific DNA binding
	SMAD3	SMAD family member 3	−2.6	2.17E−03	−0.1	2.33E−03	RNA polymerase II activating transcription factor binding
	ATXN7L3	Ataxin 7 like 3	−2.1	1.53E−02	−0.3	1.14E−02	Transcription activator activity
12	IRF2	Interferon regulatory factor 2	−8.7	2.87E−07	−1.2	1.57E−02	Transcriptional activator activity
	AHR	Aryl hydrocarbon receptor	−7.6	3.08E−06	−1.8	3.30E−03	DNA binding transcription factor activity
	TAF9	TATA-box binding protein associated factor 9	−6.5	1.36E−06	−2.0	3.98E−04	RNA polymerase II core promoter sequence-specific binding
	ATOH8	Atonal bHLH transcription factor 8	−5.8	3.11E−05	−1.3	3.54E−02	Transcription factor binding
	CARF	Calcium responsive transcription factor	−4.6	1.23E−04	−2.1	1.71E−02	Transcriptional activator activity
18	HOXB4	homeobox B4	−10.4	5.88E−08	−2.4	2.58E−02	DNA binding transcription factor activity
	AHR	Aryl hydrocarbon receptor	−9.2	4.11E−07	−1.5	1.62E−03	DNA binding transcription factor activity
	LHX2	LIM homeobox 2	−7.9	4.27E−07	−2.7	2.89E−04	Transcription factor activity
	TBX3	T-box 3	−6.5	5.68E−06	−1.4	3.26E−03	RNA polymerase II activating transcription factor binding
	LMO2	LIM domain only 2	−5.7	1.35E−06	−0.8	1.25E−02	RNA polymerase II transcription factor activity

^a^FC: fold change in expression levels relative to those of uninfected cells.

To validate the microarray results, qRT-PCR was performed to examine the expression levels of the 12 selected genes. The results show that the mRNA expression levels of six selected up-regulated genes in rSS1GFP-infected cells were much higher than that in rSS1GFP-M/NLSm-infected cells, and most gene expression levels were continuously increased from 6 to 18 hpi (Figure 5C). By contrast, the mRNA expression levels of six selected down-regulated genes in rSS1GFP-infected cells were relatively lower than that in rSS1GFP-M/NLSm-infected cells (Figure 5D). The corresponding changes were consistent with the microarray results. Taken together, we speculated that nuclear localization of NDV M protein inhibited host cell transcription by regulating the expression levels of cellular transcription regulator activity-related genes, which might be responsible for the differences in viral RNA synthesis and viral replication.

### Knockdown of PROX1 reduces viral RNA synthesis and viral replication

**Figure 6:**
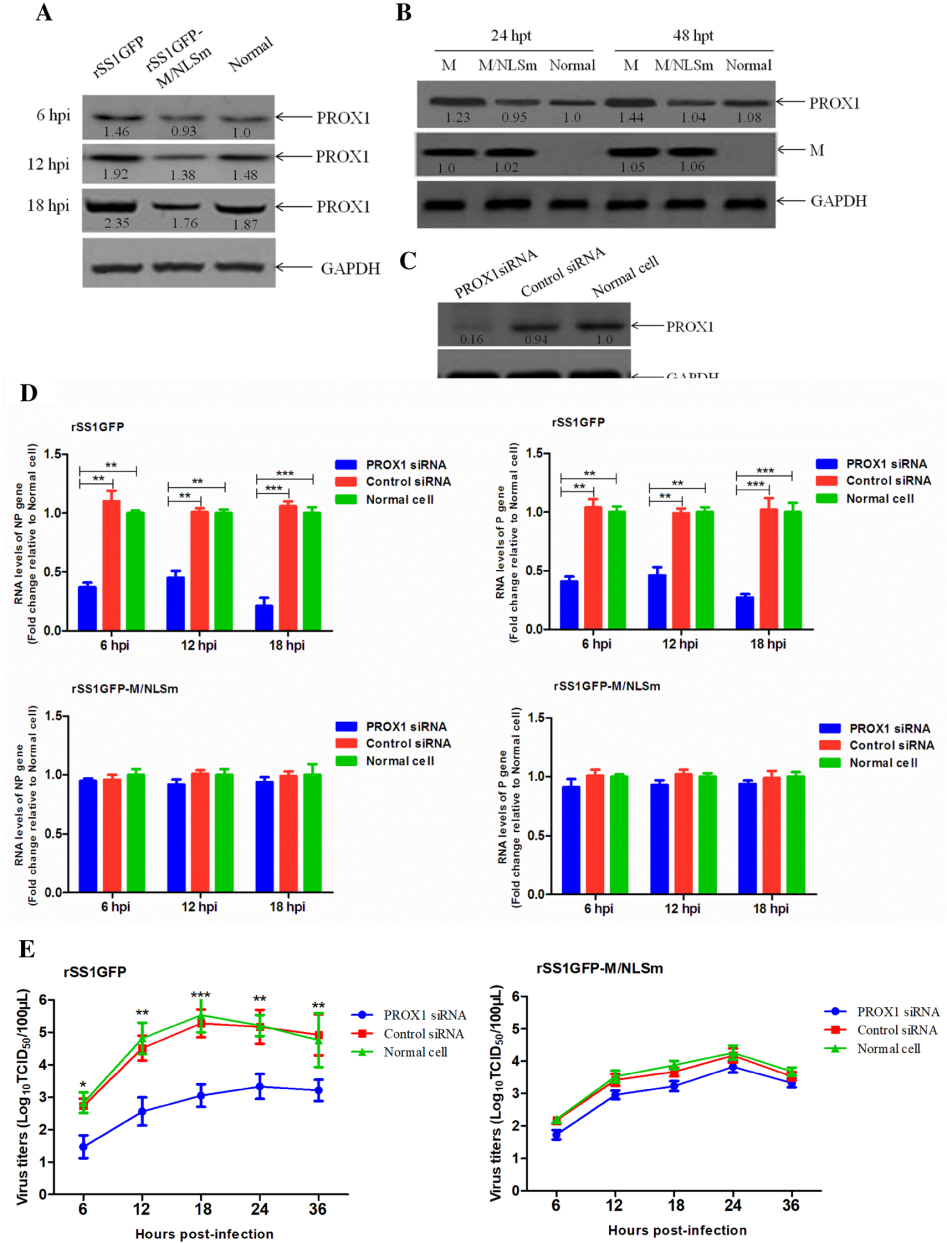
**Effect of PROX1 knockdown on the viral RNA synthesis and viral replication.** The expression levels of PROX1 protein in BSR-T7/5 cells infected with rSS1GFP and rSS1GFP-M/NLSm (**A**) or transfected with pCI-M and pCI-M/NLSm (**B**) were examined by Western blotting. The relative levels of the PROX1 protein were compared with the control GAPDH expression. **C** Effect of the PROX1 siRNA or control siRNA on the expression of endogenous PROX1 in BSR-T7/5 cells. **D** PROX1 siRNA- or control siRNA-treated BSR-T7/5 cells were infected with rSS1GFP and rSS1GFP-M/NLSm, and viral RNA synthesis corresponding to the NP and P genes were detected by qRT-PCR. **E** The growth kinetics of rSS1GFP and rSS1GFP-M/NLSm were compared using multicycle growth curves in PROX1 siRNA- or control siRNA-treated cells. Error bars represent standard deviations (mean ± SD) (*P < 0.05; **P < 0.01; ***P < 0.001 compared to the value of rSS1GFP-M/NLSm).

PROX1 is reported to be a homeodomain transcription factor essential for the development of a variety of organs, including the lymphatic system [31], the liver [32], the brain [33], and the heart [34]. In addition, a previous study has demonstrated that Kaposi’s sarcoma herpes virus (KSHV) latent gene Kaposin-B can enhance the mRNA stability of PROX1 gene and cause the up-regulation of PROX1, which is essential for KSHV replication [35]. The results of microarray analysis and qRT-PCR show that rSS1GFP infection resulted in the relatively high expression level of PROX1. Therefore, the role of PROX1 in the RNA synthesis and replication of NDV was investigated. We found that the expression levels of PROX1 protein was increased either in rSS1GFP-infected or in pCI-M-transfected BSR-T7/5 cells when compared to that of PROX1 in rSS1GFP-M/NLSm-infected or pCI-M/NLSm-transfected cells or normal cells (Figures 6A and B). To better understand the effect of PROX1 up-regulation on viral RNA synthesis and replication, siRNA-mediated knockdown of PROX1 in BSR-T7/5 cells infected with rSS1GFP was investigated. Western blotting analysis confirmed that the expression of PROX1 was significantly decreased after transfection with PROX1 siRNA (Figure 6C). Meanwhile, knockdown of PROX1 reduced the relative levels of viral genomic RNA in rSS1GFP-infected cells at different time points, but the viral RNA levels in rSS1GFP-M/NLSm-infected cells were almost not affected (Figure 6D). Moreover, the virus titers of rSS1GFP in PROX1 siRNA-treated cells was much lower than that in control siRNA-treated cells, whereas the virus titers of rSS1GFP-M/NLSm was not changed in either PROX1 siRNA- or control siRNA-treated cells (Figure 6E). Together with the above results, these results indicate that up-regulation of PROX1 caused by the nuclear localization of M could efficiently enhance viral RNA synthesis and viral replication.

### Knockdown of AHR increases viral RNA synthesis and viral replication

**Figure 7:**
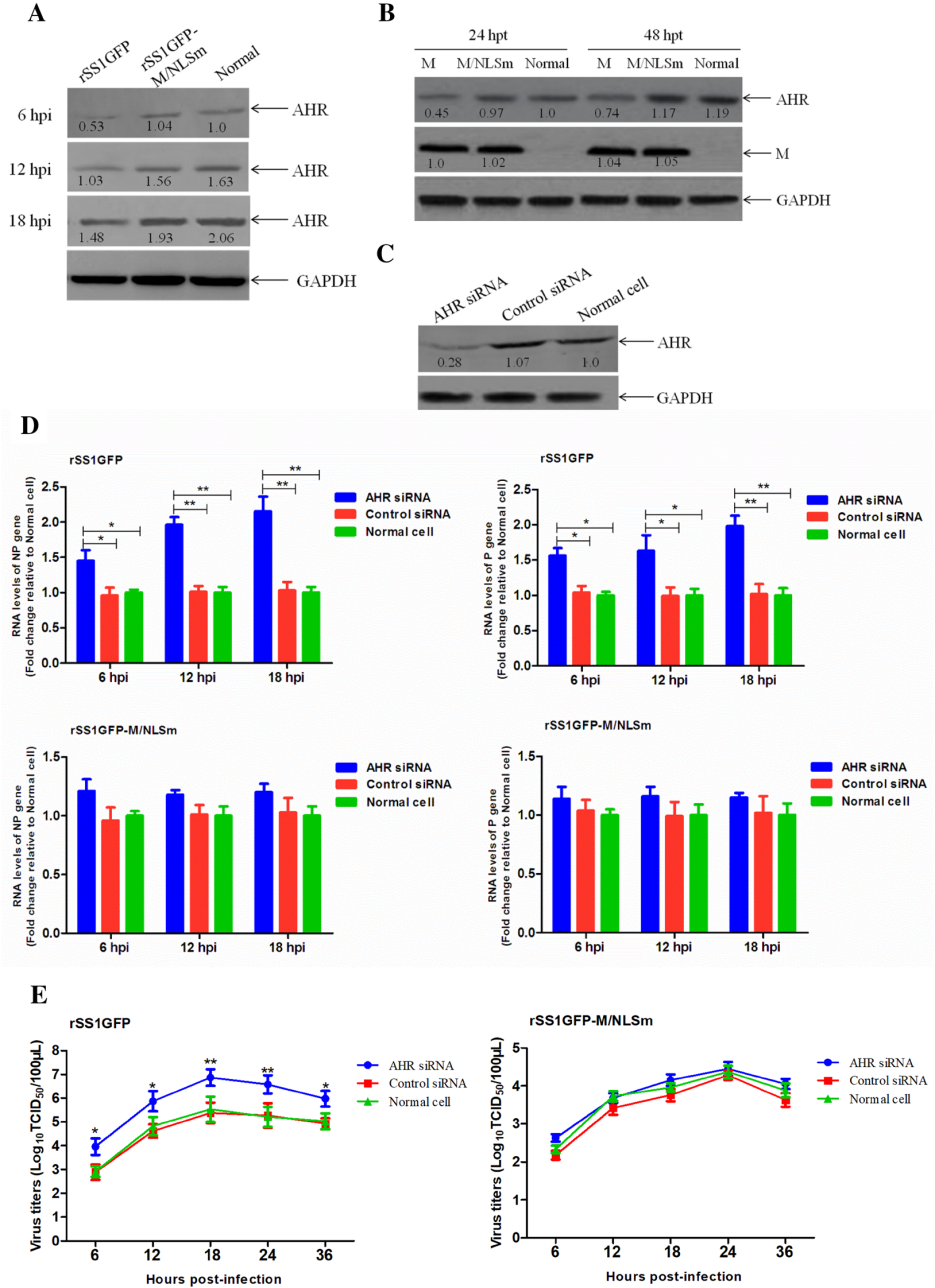
**Effect of AHR knockdown on the viral RNA synthesis and viral replication.** The expression levels of AHR protein in BSR-T7/5 cells infected with rSS1GFP and rSS1GFP-M/NLSm (**A**) or transfected with pCI-M and pCI-M/NLSm (**B**) were examined by Western blotting. The relative levels of the AHR protein were compared with the control GAPDH expression. **C** Effect of the AHR siRNA or control siRNA on the expression of endogenous AHR in BSR-T7/5 cells. **D** AHR siRNA- or control siRNA-treated BSR-T7/5 cells were infected with rSS1GFP and rSS1GFP-M/NLSm, and viral RNA synthesis corresponding to the NP and P genes were detected by qRT-PCR. **E** The growth kinetics of rSS1GFP and rSS1GFP-M/NLSm were compared using multicycle growth curves in AHR siRNA- or control siRNA-treated cells. Error bars represent standard deviations (mean ± SD) (*P < 0.05; **P < 0.01; ***P < 0.001 compared to the value of rSS1GFP-M/NLSm).

Aryl hydrocarbon receptor is a ligand-activated transcription factor, whose activation induces the expression of numerous genes and modulates host responses against viral infection [36, 37]. It is reported that AHR activation transcriptionally represses cyclin-dependent kinase (CDK) 1/2 and their associated cyclins, thereby reducing cellular dNTP levels and both HIV-1 and HSV-1 replication [38]. Interestingly, the mRNA expression levels of AHR in rSS1GFP-infected cells showed a decreasing tendency at different time points, indicating that NDV replication needed the inactivation of AHR. Therefore, we first studied the role of AHR down-regulation in viral RNA synthesis and replication of NDV. Here, we found that the protein expression levels of AHR in rSS1GFP-infected or pCI-M-transfected cells were obviously decreased in comparison to that of AHR in rSS1GFP-M/NLSm-infected or pCI-N/NLSm-transfected cells or normal cells (Figures 7A and B). We next evaluated the impact of AHR down-regulation on viral RNA synthesis and viral replication. siRNA-mediated knockdown of AHR shows that the expression level of AHR was significantly reduced in BSR-T7/5 cells when treated with AHR siRNA (Figure 7C). Accordingly, the viral RNA levels in rSS1GFP-infected cells were obviously increased at different time points, but the viral RNA levels in rSS1GFP-M/NLSm-infected cells were nearly not affected (Figure 7D). In addition, the virus titers of rSS1GFP in AHR siRNA-treated cells was much higher than that in control siRNA-treated cells and normal cells, whereas both AHR siRNA- and control siRNA-treated cells had almost no effect on the virus titers of rSS1GFP-M/NLSm (Figure 7E). Together, the above results demonstrate that down-regulation of AHR caused by the nuclear localization of M benefitted viral RNA synthesis and viral replication.

## Discussion

With the co-evolution with their hosts for many years, viruses have developed very sophisticated strategies to hijack cellular factors that promote viral uptake, replication and spread [39, 40]. Numerous reviews are now describing the interactions of various types of viral proteins with nuclear/nucleolar components, which take advantage of this specific localization to usurp one or more of its functions [41–43]. Like most cytoplasmic negative-sense RNA viruses, despite the paramyxoviruses complete viral RNA synthesis and replication in the cytoplasm, some viral proteins are localized in the nucleus at early stages of virus infection, such as the M protein of NiV, SeV, NDV, MeV and MuV, or the NP protein of MeV [5]. Up to now, several lines of evidence have demonstrated that the M protein of paramyxoviruses is a nucleocytoplasmic trafficking protein and plays essential roles in the virus life cycle [3, 5, 44]. It is noteworthy that the nuclear localization function of NNSV M protein has only been reported in HRSV [8], VSV [9, 10], and paramyxovirus MeV [11], showing the capability of inhibiting host cell transcription independently of other viral components. As an important member of the NNSV, nuclear localization of NDV M protein is thought to alter some nuclear components required for the synthesis of host transcripts, because NDV is the most effective paramyxovirus at inhibiting the production of host proteins [45]. However, the exact effect of M protein in the nucleus on the cellular nuclear functions and the replication of NDV still remains unclear.

In our studies, we first examined the dynamic changes of the intracellular localization of M for rSS1GFP and rSS1GFP-M/NLSm. In contrast to the nucleocytoplasmic shuttling of rSS1GFP M protein, the M protein of rSS1GFP-M/NLSm localized primarily around the nucleus early in infection and then distributed diffusely in the cytoplasm later in infection (Figures 1A and B). The reason for this localization pattern changes in M/NLSm protein might be that the M/NLS mutation disrupted importin β1-mediated nuclear import of the M protein [22] and caused the localization of M protein around the nucleus at 6 hpi. Meanwhile, due to the interactions of M-HN and M-NP and the role of M protein in virion assembly and budding, more M/NLSm protein might remain and distribute diffusely in the cytoplasm at 12 and 18 hpi. It has been shown that NLS-mediated nuclear localization of viral protein plays crucial roles in regulating viral replication and in evading host immunity. For example, NLS mutation in the core protein of Japanese encephalitis virus (JEV) reduces both the virus replication in mammalian cells in vitro and the pathogenesis of encephalitis induced by JEV in vivo [46]. Similarly, nuclear localized influenza A virus (IAV) nucleoprotein N-terminal deletion mutant is deficient in viral mRNA translation and exhibits a defect in functional viral ribonucleoprotein formation, which causes a delay in the replication of IAV infected cells [47]. In addition, recent studies have also indicated that nuclear import of rabies virus P protein is beneficial for inhibiting host gene transcription, regulating viral genome replication and transcription, and disrupting antiviral signaling pathways [48, 49]. Correspondingly, we previously demonstrated that NLS mutation in the M protein not only impairs the replication efficiency and plaque formation ability of NDV in DF-1 cells, but also attenuates the replication and pathogenicity of NDV in SPF chickens [22]. Here, we also found that M/NLS mutation markedly reduced both the cytopathogenicity and the syncytium formation of NDV (Figures 1C and D). There were two possible explanations for this. One reason was that rSS1GFP replicated faster and led to faster F protein expression, which directly caused cell fusion. Another reason was that the rSS1GFP-M/NLSm M protein was cytoplasmic, where it could bind to F cytoplasmic tails and restrict its fusion activity, while the rSS1GFP M protein was nuclear and separated from F protein at the early time points. Together, these findings indicated that nuclear targeting of M protein could be a key step in the replication and pathogenicity of NDV.

Based on the findings from NNSV such as HRSV, VSV and SeV, the M protein is demonstrated to inhibit transcriptase activity through M–NP interaction early in infection, and thereby repress the signal to switch from transcription to packaging into the virion particle [8, 50, 51]. Supporting this conclusion is the fact that SeV M protein can be cross-linked to the NP protein in new generated virions [52], and the addition of M protein to VSV and SeV nucleocapsids decreases their ability to transcribe viral RNA [50, 53]. The NDV M protein is shown to directly interact with viral HN and NP proteins, which are responsible for the incorporation of HN and NP proteins into virus-like particles [6]. Interestingly, we also found that M/NLS mutation reduced the viral RNA synthesis and transcription efficiency either in the minigenome assays or in viruses-infected cells (Figures 2 and 3), indicating that precocious cytoplasmic M protein had a negative effect on viral RNA synthesis and transcription. Because the NNSV M protein has the ability to inhibit viral transcriptase activity of the nucleocapsid prior to packaging and to mediate the association of the nucleocapsid with the nascent viral envelope in the later stages of virus infection [54, 55], we concluded that early accumulation of NDV M protein in the nucleus might ensure that viral RNA replication and transcription in the cytoplasm proceeded smoothly until a certain level of viral RNA and protein expression was reached, at which point the M protein could then be transported into the cytoplasmic and cell membrane to associate with the nucleocapsids for virus assembly and budding.

Numerous studies have revealed that the host response contributes to viral replication and pathogenesis [56–60]. Thus, we further elucidated the contribution of host transcriptional response to the difference in cells infected with rSS1GFP and rSS1GFP-M/NLSm. The global gene expression profile indicates that rSS1GFP greatly influenced host response, as evidenced by a larger amount of differentially expressed genes involved in binding, catalytic activity, transcription regulator activity, molecular function regulator and transporter activity (Figure 4). Functional and canonical analysis using IPA reveals that rSS1GFP actively motivated virus infection-induced signaling pathways (Table 2). Of all these pathways, rSS1GFP infection obviously caused the signaling pathway of transcriptional misregulation at 12 and 18 hpi (Additional file 2), suggesting that nuclear localization of M protein affected the process of host cell transcription. Remarkably, one of the most important findings of our study was that nuclear localization of NDV M protein inhibited host cell transcription, showing that most up-regulated genes were associated with transcription repressor activity and negative regulation of transcription, whereas the down-regulated genes were involved in DNA binding or RNA polymerase II transcription factor activity and transcriptional activator activity (Figure 5, Tables 3 and 4). For the investigation of nuclear localization functions of NNSV M protein, a previous study has shown that nuclear extracts from HRSV-infected cells have less transcriptional activity in vitro and inhibit the transcriptional activity of nuclear extracts from mock-infected cells, suggesting that HRSV M protein plays a role in inhibiting host cell transcription [28]. In addition, a recent study has demonstrated that transient expression of MeV M protein in transfected cells inhibits cellular transcription via binding to nuclear factors [11]. Additionally, the M protein is able to inhibit in vitro transcription in a dose-dependent manner, indicating the role of MeV M in inhibiting host cell transcription [11]. Moreover, studies focusing on VSV M protein revealed that M protein directly inhibits host cell transcription by inactivating essential transcription factors [9], and also inhibits cellular nuclear transport to impair mRNA export, indirectly leading to a decrease and an increase in host cell and virus transcription [30, 61, 62]. Based on the results of microarray analysis, we speculated that NDV M protein possibly hijacked the expression of transcription regulator activity related genes to affect host mRNA synthesis, which in turn effected the inhibition of host cell transcription. Together, the above findings suggest that the inhibition of host cell transcription caused by the nuclear localization of M in NNSV could occur through diverse pathways.

**Figure 8:**
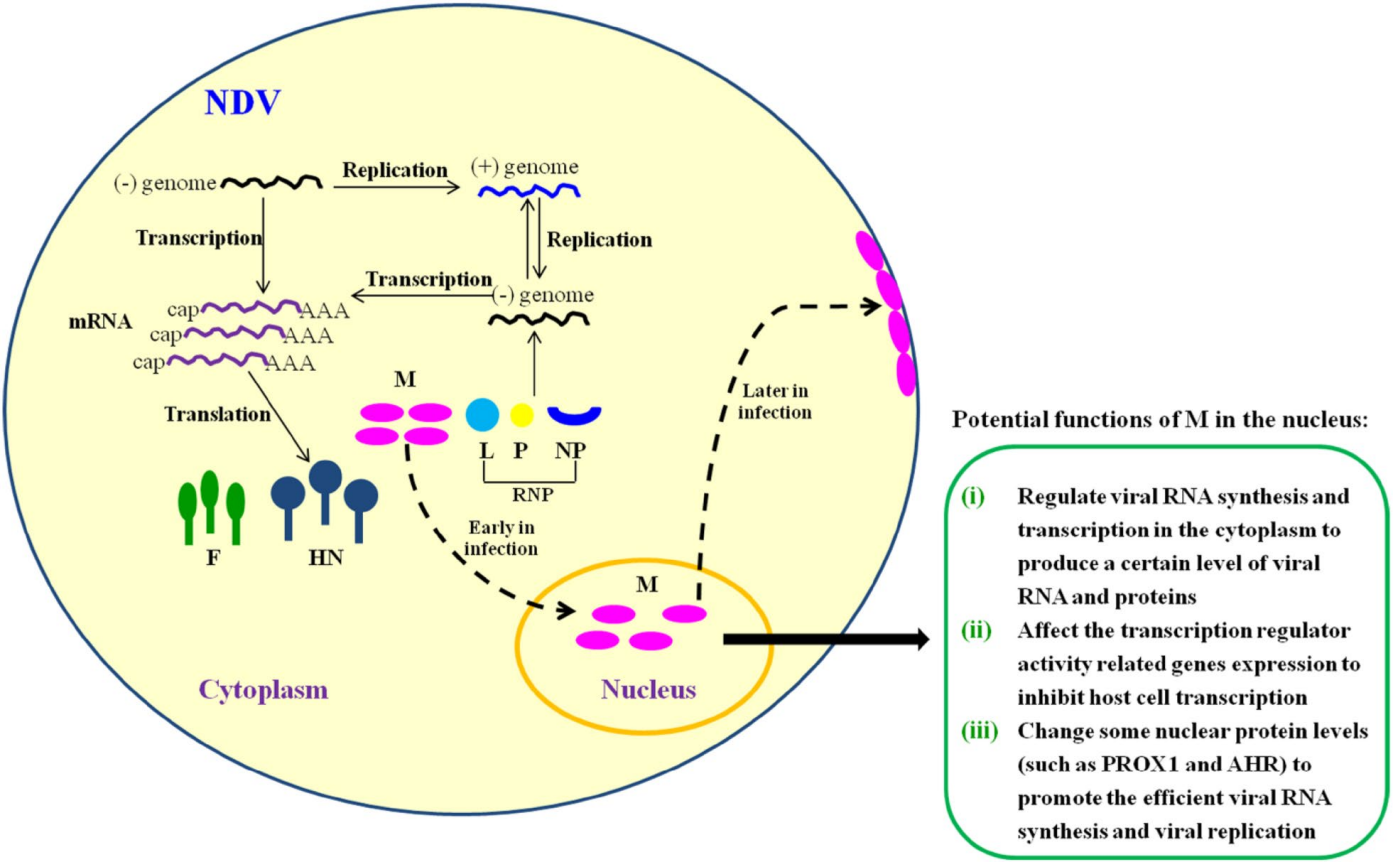
**The schematic diagram of the replication and transcription of NDV genome and the potential functions of M protein in the nucleus.** Transcription and replication of the NDV genome occurs in the cytoplasm by the action of viral RNP. During the course of NDV infection, the M protein is localized in the nucleus early in infection and enters the cytoplasm and binds to the cellular membrane later in infection. The potential functions of M in the nucleus are indicated according to our findings.

It is notable that virus-host interactions will greatly improve our understanding of the replication and pathogenesis of viruses. Therefore, we further investigated the role of the selected up-regulated gene PROX1 and down-regulated gene AHR in the viral RNA synthesis and replication of NDV. It is reported that PROX1 and AHR genes are a kind of transcription factors that play essential roles in cell life activities, and more importantly, the up-regulation of PROX1 or down-regulation of AHR has been reported to be beneficial for virus replication found in KSHV [35] or HIV-1 and HSV-1 infection [38]. In our studies, we also found that the expression levels of PROX1 and AHR proteins were obviously increased or decreased in rSS1GFP-infected cells or pCI-M-transfected cells (Figures 6A, B and 7A, B), which was consistent with the results of qRT-PCR (Figures 5C and D). Remarkably, cells transfected to express viral NP or HN protein had no effect on the expression levels of PROX1 and AHR (data not shown), indicating that nuclear localization of M protein played important roles in regulating this process. In addition, siRNA-mediated knockdown of PROX1 or AHR significantly reduced or increased the viral RNA levels and virus replication ability (Figures 6D, E and 7D, E), respectively, suggesting the important roles of the expression level changes of PROX1 and AHR in the NDV life cycle. However, how M protein affects the expression of PROX1 and AHR and what the specific roles of PROX1 and AHR in the replication of NDV are currently unknown. Further investigation into the signaling pathways that are involved in their interactions and functions are needed to gain a better understanding of the underlying replication of NDV. In summary, our findings reveal for the first time the potential functions of NDV M protein in the nucleus (Figure 8), which will provide foundations for investigating the role of M protein in the replication and pathogenesis of NDV and other paramyxoviruses.

## Additional files

**Additional file 1.** **Schematic representation of viral RNA synthesis and GFP reporter gene translation in the minigenome system.** BSR-T7/5 cells were co-transfected with a minigenome plasmid pTVT-LGT, helper plasmids pCI-NP, pCI-P, pCI-L and pCI-M or pCI-M/NLSm. Antigenomic, positive-sense minigenome RNA (gRNA[+]) was transcribed from the minigenomic plasmid by T7 RNA polymerase. In the presence of NP, P and L, gRNA(+) acted as a template for the transcription of genomic RNA (gRNA[-]), which generated GFP reporter gene mRNA and more gRNA(+).

**Additional file 2.** **Modeling of the signaling pathway of transcriptional misregulation in DF-1 cells infected with rSS1GFP at 12 hpi (A) and 18 hpi (B).** The signaling pathway of transcriptional misregulation induced by rSS1GFP infection was drawn, and the significantly differentially expressed genes involved in this pathway are indicated: a red label indicates up-regulated genes; a green label indicates down-regulated genes.
