# Supplementary figures and images for: Nuclear localization of Newcastle disease virus matrix protein promotes virus replication by affecting viral RNA synthesis and transcription and inhibiting host cell transcription
Source: Vet Res. 2019 Mar 20;50:22. doi: 10.1186/s13567-019-0640-4 (PMC6425612; doi:10.1186/s13567-019-0640-4)

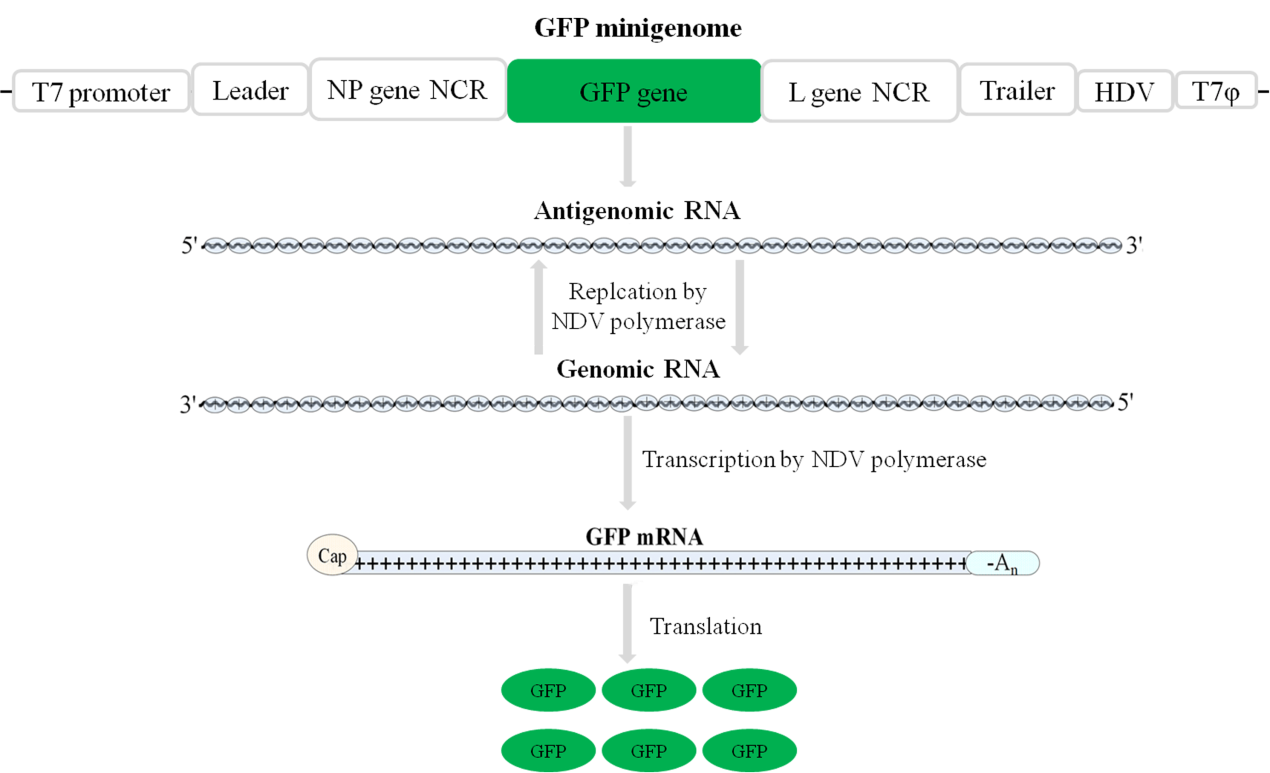

Supplement: Supplementary file 1 — Additional file 1. Schematic representation of viral RNA synthesis and GFP reporter gene translation in the minigenome system. BSR-T7/5 cells were co-transfected with a minigenome plasmid pTVT-LGT, helper plasmids pCI-NP, pCI-P, pCI-L and pCI-M or pCI-M/NLSm. Antigenomic, positive-sense minigenome RNA (gRNA[+]) was transcribed from the minigenomic plasmid by T7 RNA polymerase. In the presence of NP, P and L, gRNA(+) acted as a template for the transcription of genomic RNA (gRNA[-]), which generated GFP reporter gene mRNA and more gRNA(+). [file 13567_2019_640_MOESM1_ESM.doc]

**A** 12 hpi


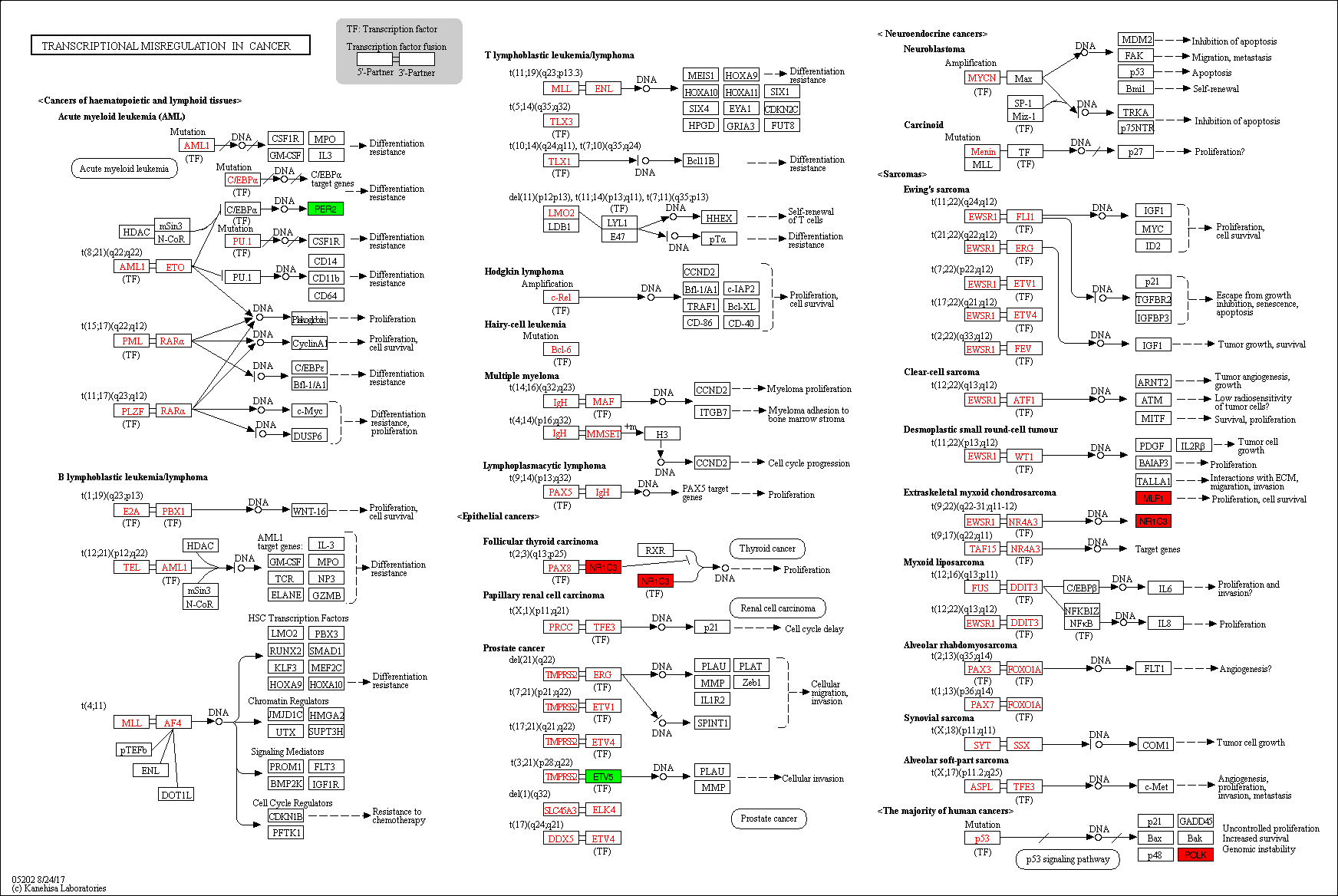


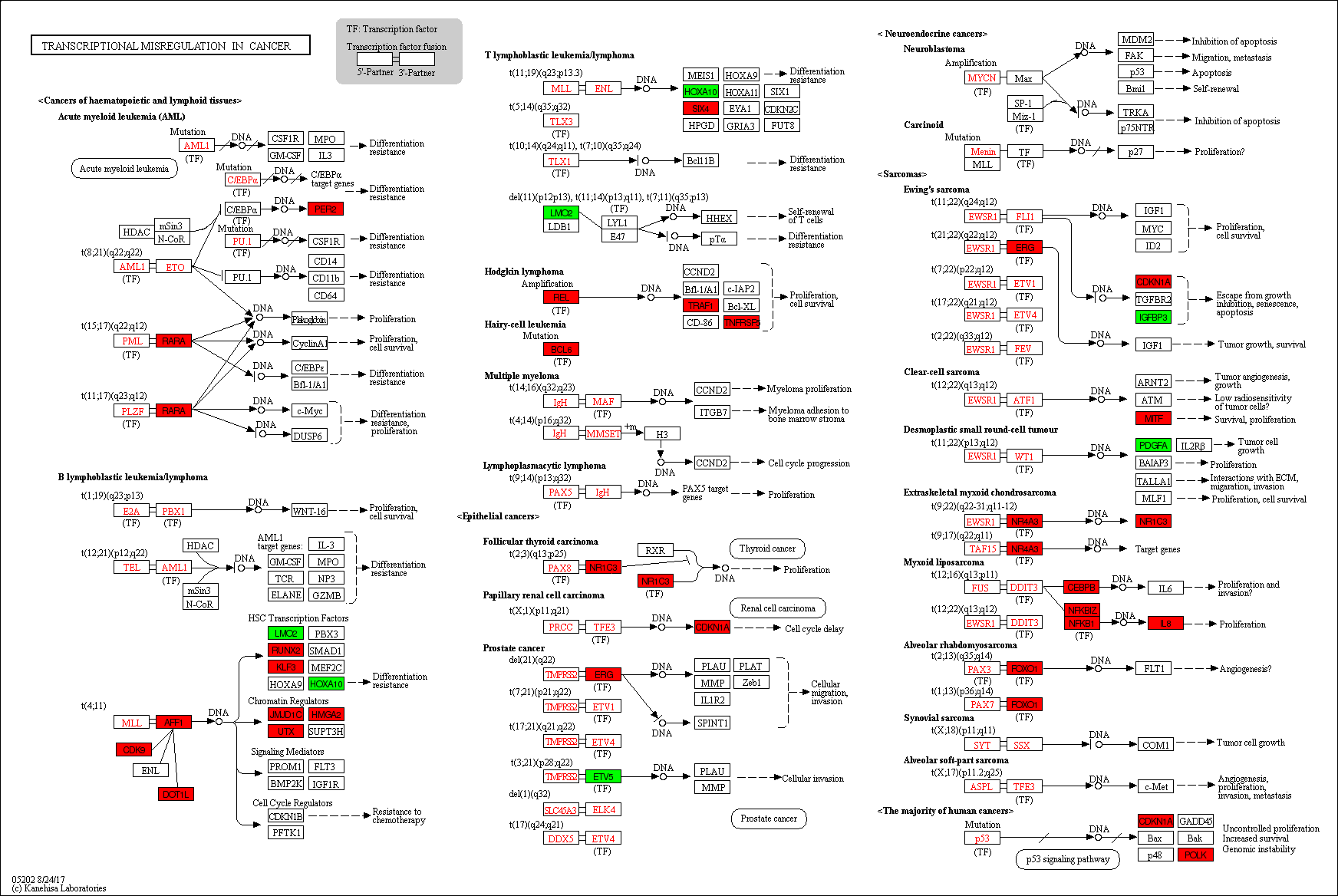


**B** 18 hpi

Supplement: Supplementary file 2 — Additional file 2. Modeling of the signaling pathway of transcriptional misregulation in DF-1 cells infected with rSS1GFP at 12 hpi (A) and 18 hpi (B). The signaling pathway of transcriptional misregulation induced by rSS1GFP infection was drawn, and the significantly differentially expressed genes involved in this pathway are indicated: a red label indicates up-regulated genes; a green label indicates down-regulated genes. [file 13567_2019_640_MOESM2_ESM.docx]
